# Molecular Aspects of Cardiovascular Risk Factors

**DOI:** 10.3390/biom14081032

**Published:** 2024-08-20

**Authors:** Shang-Zhong Xu, Thozhukat Sathyapalan

**Affiliations:** 1 Centre for Atherothrombosis and Metabolic Disease, Hull York Medical School, University of Hull, Hull HU6 7RU, UK; 2 Academic Endocrinology, Diabetes and Metabolism, Hull York Medical School, University of Hull, Hull HU6 7RU, UK

Cardiovascular diseases (CVDs) are the leading cause of death. Around 20.5 million people died from CVDs in 2021, which accounts for 32% of all causes of death [1,2]. Over three-quarters of CVD-related deaths occur in low- and middle-income countries, and 85% of CVD deaths are due to heart attacks and strokes [3,4]. The economic burden of CVDs is expected to increase substantially in the coming decades [5,6]. Therefore, a deep understanding of the molecular aspects of CVDs and development of cost-effective programmes and policies to control cardiovascular risk factors are urgently needed.

There are many risk factors for CVDs, including modifiable risk factors, such as behavioural and environmental risk factors (unhealthy diet, physical inactivity, alcohol abuse, smoking, and air pollution); social risk factors (anxiety, sleep disorders, psychological distress, and poverty); some comorbidities (diabetes, hypertension, hyperlipidaemia, obese or overweight, chronic kidney disease, inflammation, and infection); and non-modifiable risk factors, such as genetic variations with family history, ethnicity, gender, and ageing (Figure 1). Multiple risk factors such as these trigger various molecular targets and eventually result in endothelium dysfunction and remodelling in the blood vessels and heart.

Most CVDs or cardiovascular events can be prevented by controlling modifiable risk factors and comorbidities, particularly premature coronary artery disease (CAD), which occurs in males younger than 45 years and females younger than 55 years. The American Heart Association (AHA) has introduced Life’s Essential 8 to assess cardiovascular health with a scoring algorithm ranging from 0 to 100 points [7]. The eight essential components include a healthy diet, physical activity, the avoidance of nicotine, healthy sleep, a healthy weight, healthy levels of blood lipids, blood glucose, and blood pressure. These components are core health behaviour factors (smoking, physical activity, nutrition, sleep, and obesity) and health factors (cholesterol, blood pressure, and glucose control) that mainly contribute to the risk of cardiovascular health. These factors are modifiable or manageable via lifestyle changes and the proper use of medicine during the life course. It has been shown through longitudinal monitoring that the implementation of cardiovascular health measures can help to reduce cardiovascular risk and cardiovascular events and mortality [8].

This Special Issue showcases three research articles and six reviews on the molecular targets of cardiovascular risk factors and the biomarkers and signalling pathways of the pathophysiological aspects of CVDs.

Ca^2+^ is a central player in the cardiovascular system, with functions including the regulation of cell functions (contraction, secretion, migration, growth, and death) and tissue calcification. Ca^2+^ influx and efflux across the cell membrane are strictly controlled by Ca^2+^ channels and transporters. Among them, voltage-gated Ca^2+^ channels have been well described as therapeutic drug targets in the cardiovascular system. Over the last two decades, the importance of several Ca^2+^-permeable channels and/or cationic channels has also been demonstrated as molecular targets for cardiovascular diseases, such as the TRP channel family, ORAI channels, and the mechanically sensitive cation channels of the PIEZO family [9,10]. TRPC1 was first identified in vascular smooth muscle cells in 2001 as a store-operated channel subunit [11]. The upregulation of TRPC1 was seen in proliferative smooth muscle cells and led to the formation of neointimal growth and arterial restenosis [12]. TRPC1 also interacts with TRPC5 to form heteromultimeric channels (TRPC1/5); The TRPC1/5 channel can be directly activated by thioredoxin and is involved in the pathogenesis of rheumatoid arthritis [13]. In the study by Chen et al., it is found that TRPC4 and TRPC5 are new targets of homocysteine. Their channel activities are regulated by homocysteine and Cu^2+^ complexes at the binding sites of glutamic acids (E542/E543) and the cysteine residue (C554) in the extracellular pore region of TRPC4 [14]. An elevated level of circulating homocysteine has been regarded as an independent risk factor for CVDs; the regulation of TRPC channels by homocysteine is therefore a novel mechanism for the pathogenesis of CVDs in hyperhomocysteinemia.

Vascular inflammation and calcification are common pathophysiological processes in atherosclerosis and show a strong correlation with cardiovascular events [15,16]. The molecular link between vascular inflammation and calcification is still unclear. Macri et al. demonstrated that the JAK-STAT signalling pathway connects the inflammatory molecules to calcium deposition in vascular smooth muscle cells and aortic tissue [17]. The authors showed that the blocking of the JAK-STAT cascade reduces smooth muscle cell proliferation and pro-inflammatory factor expression and release while increasing calcium deposition and the expression of the osteogenic transcription factor RUNX2 [17]. The clinical benefits of potential anti-inflammatory therapeutics for CVDs are still a subject of debate. There are some reports showing that the blockage of inflammatory pathways and cytokines can ameliorate cardiovascular events, such as the use of colchicine or inhibitors of tumour necrosis factor, but none of the clinical studies has confirmed cardioprotective effects [18]. On the other hand, statins have anti-inflammatory effects and reduce cardiovascular events but concurrently promote coronary artery calcification [19].

Smoking is a traditional risk factor for CVDs [20]. Cigarette and e-cigarettes contain nicotine, which causes an overproduction of reactive oxygen species (ROS) and a reduction in endothelial nitric oxide (NO), as well as inflammation, thrombosis, and abnormal lipid metabolism [21]. However, there has been a debate about cardiovascular risk in cannabis smokers. With the increase in popularity of cannabis and the legalisation of cannabis in some countries, this public health issue is becoming a hot medical topic [22]. For their article, Shan and co-workers investigated the role of cannabinoid type 2 receptor (CB2) in neuroinflammation and hypertension using an in vitro cell model and in vivo mouse models and demonstrated that the activation of CB2 can reduce blood pressure and neuroinflammation. These effects could be beneficial in potential medical use [23]. A more rigorous clinical evaluation of the potential therapeutic use of cannabis and cannabinoids, or personal use for health effects, is necessary before future public health regulation frameworks can be implemented.

The renin–angiotensin–aldosterone system (RAAS) is an important signalling pathway for the regulation of cardiovascular functions. Many therapeutic drugs have been successfully developed to target this signalling pathway, including angiotensin-converting enzyme inhibitors (ACEIs) and angiotensin receptor blockers, which are widely used in the clinic to treat hypertension and heart failure. The review by Crompton et al. is focused on the steroid hormone aldosterone and goes into detail on its pathophysiological roles in promoting atherosclerosis, inflammation, and the permeability of the vascular endothelium and glomerular filtration barrier [24]. Pharmacological interventions using aldosterone and its mineralocorticoid receptor are also addressed in this review article [24].

Sphingosine-1-phosphate (S1P) is a signalling sphingolipid in the body and regulates cell proliferation, migration, differentiation, and apoptosis in the cardiovascular and immune systems [25]. The effects of S1P are mainly mediated by the G protein-coupled receptors of SIP (S1PR1, S1PR2, and S1PR3), which are expressed in vascular endothelial cells, smooth muscle cells, cardiomyocytes, and fibroblasts. The signalling of SIP is also linked to Ca^2+^ channels, since it has been shown to directly activate TRPC5 channels and TRPC5–TRPC1 heteromultimeric channels via two mechanisms (one extracellular and one intracellular) and increase vascular smooth muscle cell motility [25]. The review by Wang et al. summarises its physiological and pathological roles in the cardiovascular system [26]. The signalling pathways and the roles of S1P in atherosclerosis, angiogenesis, hypertension, and myocardial ischemia are comprehensively presented in this review [26].

Unhealthy diet and physical inactivity are modifiable risk factors. Abraham et al. summarise epigenetic modifications via these two behavioural risk factors [27]. The authors include evidence of the effects of diet or diet supplements and physical exercise on DNA methylation. The underlying signalling pathways and associated biomolecules for the epigenetic profile changes in metabolic disorders are reviewed. The authors suggest that lifestyle change can restore the epigenetic profile and reduce the risk for cardiovascular and metabolic disorders.

Large interventional studies have suggested that hyperglycaemia, and poor glycaemic control resulting in hypoglycaemia, are largely responsible for the development of CVDs [28]. Besides the hyperglycaemia condition in diabetes, hypoglycaemia that has been previously neglected has now been demonstrated to be a risk factor for CVDs in several large clinical trials. The pathophysiological mechanisms include enhanced coagulation, oxidative stress, vascular inflammation, endothelial dysfunction, and platelet activation [29]. The review by Ali et al. in this Special Issue summarises the recent understanding of abnormal functionality in platelets under hypoglycaemia conditions and highlights therapeutic strategies for preventing CVDs by reducing the HbA1c level but avoiding hypoglycaemia [30].

Heart failure (HF) is a major global health concern, with around 56.2 million people living with this disease [2]. The main conditions that lead to heart failure include coronary heart disease, hypertension, cardiomyopathy, atrial fibrillation, heart valve disorders, and congenital heart diseases. In this Special Issue, Kryczka et al. present a review on peripartum cardiomyopathy, a specific group of heart failure types, which is a severe cardiovascular condition seen during pregnancy or over several weeks following delivery [31]. The authors go into detail on the pathophysiological aspects and biomarkers of peripartum cardiomyopathy. The application of bromocriptine could be a specific treatment for peripartum cardiomyopathy following delivery, as it inhibits the secretion of prolactin from the pituitary gland and improves cardiac function.

Chronic kidney disease (CKD) represents one of the strongest risk factors for CADs and particularly for heart failure [32]. The underlying mechanisms behind the link between kidney disease and CVDs could be due to the accumulation of toxic metabolites, hyperuricemia, albuminuria, anaemia, oxidative stress, inflammation, endothelial dysfunction, and some shared pathophysiology of comorbidities (diabetes, hypertension, and dyslipidaemia). The specific treatment options for patients with advanced CKD are very limited, but the application of sodium-glucose cotransporter-2 (SGLT2) inhibitors has recently been shown to offer not only a glucose-lowering effect in patients with diabetes, but also significant cardiovascular and kidney-protective effects [33]. The protective mechanisms of SGLT2 inhibitors are still unclear. The reduction in cytosolic and mitochondrial ROS enabled by the SGLT2 inhibitor dapagliflozin could represent one explanation for its protective effects [34].

Precisely monitoring kidney function is important in disease prevention and the prediction of prognosis for follow-up studies. The eGFR is a commonly used index for kidney filtration function. In this Special Issue, Spencer et al. summarise the clinical applications of cystatin C and creatinine for kidney function monitoring [35]. Based on accuracy and influencing factors, Cystatin C has advantages over creatinine as a marker of kidney function. The cost, availability, and accessibility of routine cystatin C testing in clinical practice are well justified.

This Special Issue on molecular aspects of cardiovascular risk factors covers a number of different risk factors; many molecules and signalling pathways are not unique to CVDs but are shared with other disease processes, for example, cellular signalling for oxidative stress and inflammation. Some risk factors, such as obesity, lipoproteins, and genetic factors, are not fully explored in this Special Issue. However, we hope that this Special Issue reflects the current awareness in this field and that the collected articles will advance our understanding of cardiovascular risk factors and strategies to prevent CVDs.

## Figures and Tables

**Figure 1:**
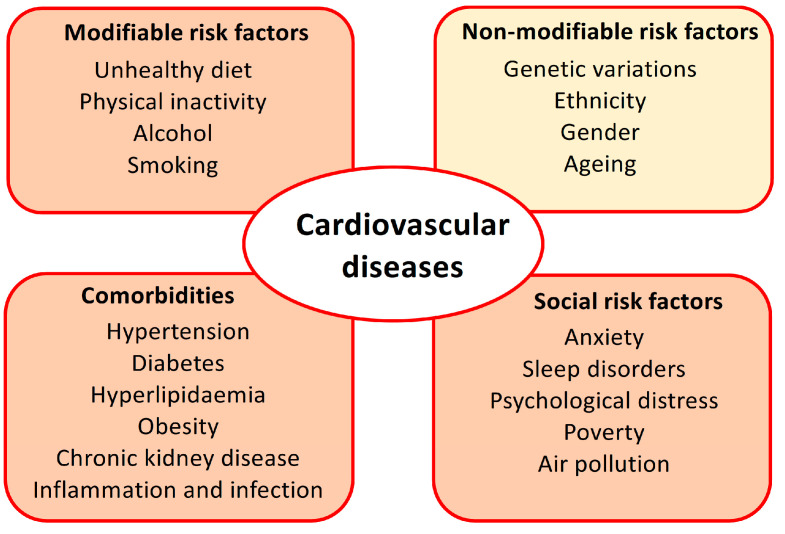
Main risk factors for cardiovascular diseases (CVDs). The modifiable behaviour risk factors, comorbidities, and social risk factors can be controlled via lifestyle changes, medical care, public health awareness campaigns, and prevention or mitigation strategies from governments and communities. The non-modifiable factor of ageing may also be influenced by an intervention with Life’s Essential 8 measures, which have been shown to slow the biological ageing process.
